# Incidents of Animal Life

**Published:** 1887-05-14

**Authors:** F. O. Morris

**Affiliations:** Rector of Nunburnholme, Yorkshire; author of "A History of British Birds," etc.


### Incidents of Animal Life.

Collated by the Rev. F. O. MORRIS, B.A.,

Rector of Nunburnholme, Yorkshire; author of "A History of British Birds," etc.

"He prayeth best, who loveth best
All things both great and small;
For the dear God who loveth us,
He made and loveth all."—Coleridge.

### Dogs and their Doings.

I read an account recently of a lecture Sir John Lubbock had been giving a short time since, in which, as the review of it would seem to imply, he appeared to think it a discovery that dogs can understand words spoken to them. But it is nothing new, as shown by the following anecdotes I take from my *Records of Animal Sagacity*, which, as it has been out of print for several years—*pari passu* with the *Anecdotes in Natural History*, of which I had to state the like the other day in The Hospital—will, I have little or no doubt, be new to most, if not to every one of your readers. Indeed, they are shown not only to understand the meaning of words spoken to them, but spoken of them, or in their hearing.

"The wisest dog I ever had," said Sir Walter Scott, "was what is called the bull-dog terrier. I taught him to understand a great many words, insomuch that I am positive that the communication between the canine species and ourselves might be greatly enlarged. Camp once bit the baker, who was bringing bread to the family. I beat him, and explained the enormity of his offence, after which, to the last moment of his life, he never heard the least allusion to the story, in whatever voice or tone it was mentioned, without getting up and retiring into the darkest corner of the room, with great appearance of distress. Then if you said, the baker was well paid, or, the baker was not hurt after all, Camp came forth from his hiding-place, capered, and barked, and rejoiced. When he was unable, towards the end of his life, to attend me when on horseback, he used to watch for my return, and the servant would tell him his master was coming down the hill, or through the moor, and although he did not use any gesture to explain his meaning, Camp was never known to mistake him, but either went out at the front to go up the hill, or at the back to get down to the moor-side. He certainly had a singular knowledge of spoken language."

Sir Walter Scott has also told a number of anecdotes of a dog called Dandie, the property of another gentleman, which knew on most occasions what was said in his presence. His master returning home one night rather late, found all the family in bed, and not being able to find the boot-jack in its usual place, said to his dog, "Dandie, I cannot find my boot-jack; search for it." The dog, quite sensible of what had been said to him, scratched at the room door, which his master opened, proceeded to a distant part of the house, and soon returned, carrying in his mouth the boot-jack, which his master had left that morning under a sofa.

That they in reality learn, say the Messrs. Chambers of Edinburgh, in their "Anecdotes of Dogs," to know the meaning of certain words, not merely when addressed to them, but when spoken in ordinary conversation, is beyond a doubt, although the accompanying looks and movements in all likelihood help them in their interpretation. We have known a small spaniel, for instance, which thoroughly understood the meaning of "out," or "going out," when spoken in the most casual way in conversation.

They add: "A lady of our acquaintance has a dog which lives at enmity with another dog in the neighbourhood, called York, and angrily barks when the word York is pronounced in his hearing."

The late Dr. J. McCulloch has related of his own knowledge, that a shepherd's dog always eluded the intentions of the household regarding him, if aught was whispered in his presence that did not coincide with his wishes.

James Hogg, in his *Shepherd's Calendar*, declares that dogs know what is said on subjects in which they feel interested. A farmer had a dog that for the space of three or four years, in the latter part of his life, met him always at the foot of his farm, about a mile and a half from his house, on his way home. If he was half a day away, a week, or a fortnight, it was all the same; she met him at that spot; and there never was an instance seen of her going to wait his arrival there on a wrong day. She could only know of his coming home by hearing it mentioned in the family.

The same writer speaks of a clever sheep-dog named Hector, which had a similar tact in picking up what was said. One day he observed to his mother, "I am going to-morrow to Bowerhope for a fortnight; but I will not take Hector with me, for he is constantly quarrelling with the rest of the dogs." Hector, who was present, and overheard the conversation, was missing next morning, and when Hogg reached Bowerhope, there was Hector sitting on a knoll, waiting his arrival. He had swum across a flooded river to reach the spot.

Still more surprising, say the Messrs. Chambers, "dogs may be trained not only to know the meaning of words, but to speak them. The learned Leibnitz reported to the French Academy that he had seen a dog in Germany which had been taught to pronounce certain words. The teacher of the animal, he stated, was a Saxon peasant boy, who, having observed in the dog's voice an indistinct resemblance to various sounds of the human voice, was prompted to endeavour to make him speak. The animal was three years old at the beginning of his instructions, a circumstance which must have been unfavourable to the object; yet, by dint of great labour and perseverance, in three years the boy had taught it to pronounce thirty German words. It used to astonish its visitors by calling for tea, coffee, chocolate, etc.; but it is proper to remark that it required its master to pronounce the words beforehand; and it never appeared to become quite reconciled to the exhibitions it was forced to make."

